# Factors associated with the decline of malaria in Myanmar’s Ayeyarwady Region between 2013 and 2017

**DOI:** 10.1038/s41598-021-99737-4

**Published:** 2021-10-14

**Authors:** Sarah Gallalee, Abigail V. Ward, Moe Moe Aye, Nang Khaing Zar Aung, Julia C. Dunn, Stephen Lavenberg, Christopher Lourenço, Jillian Dunning, Aung Thi, Arnaud Le Menach, Myat Min Tun

**Affiliations:** 1 Clinton Health Access Initiative, Inc., Boston, MA USA; 2 Myanmar Vector Borne Disease Control Program, Ministry of Health and Sports, Nay Pyi Taw, Myanmar

**Keywords:** Malaria, Epidemiology

## Abstract

The burden of malaria in Myanmar has declined rapidly in recent years; cases decreased from 333,871 in 2013 to 85,019 in 2017 (75% decrease). Decline of malaria in the Ayeyarwady Region of Myanmar reflects this trend with an 86% decrease in cases over this period. In this exploratory analysis, quantitative and qualitative information were assessed to explore potential factors responsible for the decline of malaria in Ayeyarwady. Data on malaria incidence, programmatic financing, surveillance, case management, vector control interventions, climate and ecological factors, and policies and guidelines spanning 2013 to 2017 were compiled. Poisson regression models that adjust for correlation were used to analyze the association between annual malaria case numbers with malaria intervention factors at the township level. Between 2013 and 2017, there was a decrease in mean township-level malaria incidence per 1000 from 3.03 (SD 4.59) to 0.34 (SD 0.79); this decline coincided with the implementation of the government’s multi-pronged malaria elimination strategy, an increase of approximately 50.8 million USD in malaria funding nationally, and a period of deforestation in the region. Increased funding in Ayeyarwady was invested in interventions associated with the decline in caseload, and the important roles of surveillance and case management should be maintained while Myanmar works towards malaria elimination.

## Introduction

The Greater Mekong Subregion (GMS), consisting of Cambodia, Laos, Myanmar, Thailand, Vietnam, and one province of China, has been a focal point of malaria efforts in recent years because drug resistance to the first-line treatment, artemisinin-based combination therapies (ACTs), has become widespread in the subregion and there is a high risk of resistance spreading to other malaria endemic regions^1, 2^. Although Myanmar has the highest burden of malaria in the GMS (over 50% of reported cases in the subregion were in Myanmar in 2017), the country has achieved a 75% reduction in reported cases between 2013 and 2017 (333,871 to 85,019)^3, 4^.

Myanmar aims to eliminate *Plasmodium falciparum* by 2025 and eliminate all malaria by 2030^4^. Combined efforts by the National Malaria Control Program (NMCP), the World Health Organization (WHO), the Global Fund to Fight AIDS, Tuberculosis, and Malaria (GF), private entities, and non-governmental organizations to eliminate malaria have been ongoing for decades in Myanmar, with a full package of case management and vector control interventions and guidelines implemented throughout the country^4^. Primary interventions for malaria include: long-lasting insecticide-treated net (LLIN) distributions through mass campaigns every three years in at-risk villages (i.e., villages with annual parasite incidence [API] over 0) and routinely at health facilities to high risk populations (such as forest goers and pregnant women) and to rapid diagnostic test (RDT)-positive patients; integrated community malaria volunteer (ICMV) placements in high risk villages to maximize access to diagnosis and treatment; information, education, and communication (IEC) messaging to promote LLIN use through media channels and basic health staff (BHS); and improvements to surveillance, program management, partnership, and capacity building^5^. A study recently completed in three areas of Myanmar (Kayin State, Tanintharyi Region, and Rakhine State) by the United States Agency for International Development (USAID)/ President’s Malaria Initiative (PMI) showed significant associations at the township level between declining reported cases and the following factors: insecticide-treated bed net (ITN) distribution, concentration of village health workers, amount of health worker training, and socioeconomic status^6^. Another study on the incidence of malaria in Myanmar between 2005 and 2014 found that declining malaria coincided with the increase in government malaria control program activities including deployment of volunteers, distribution of ITNs, and improving ACT availability^7^. In addition to Myanmar’s interventions for malaria elimination, changes in environmental and ecological factors may be related to decreased malaria transmission and incidence. Previous studies have examined associations between environmental variables and malaria transmission. A study in Tak Province, Thailand, identified associations of malaria cases with temperature and humidity^8^. Another recent study in the Ayeyarwady Region of Myanmar found that sleeping in the forest in the past month was a risk factor for malaria [Dunning et al., *manuscript in preparation*]. Overall, three hypotheses could explain the decline of malaria in Myanmar: the malaria control activities have worked as expected, climate and ecological factors (such as deforestation) have caused the decline regardless of the interventions, or control activities and ecological factors could have had a combined impact on malaria transmission.

Elucidating the impact of interventions and understanding which tools are most effective in certain settings have become priorities for malaria programs globally. This is especially pertinent for countries and sub-national areas targeting elimination because limited resources must be used in the most cost-effective and high-impact manner^8–10^. Understanding factors that have led to the recent reduction in malaria burden in Myanmar is critical to inform effective measures for future interventions to sustain progress and accelerate elimination efforts within the country. This study focuses on the region of Ayeyarwady in southwest Myanmar, which has experienced a rapid decline in reported cases: between 2013 and 2017, confirmed public sector cases in the region dropped from 14,099 to 1919 (an 86% decrease)^11^. Malaria elimination efforts in Ayeyarwady are now focused on the next milestone of zero indigenous cases (case contracted locally) by the end of 2025^4^. This study was a collaborative effort between the Vector Borne Disease Control (VBDC) unit in Ayeyarwady and the Clinton Health Access Initiative (CHAI). In this study we first determine the reliability of malaria case data by analyzing trends in surveillance reporting completeness and malaria testing in Ayeyarwady. After the reliability of the data is assessed we then analyze malaria epidemiology in conjunction with environmental factors to determine how factors unrelated to coordinated intervention efforts impact malaria in the region. Finally, we examine the association between malaria epidemiology data and intervention data to identify possible drivers of malaria decline in Ayeyarwady. Throughout we also consider qualitative information, gathered from the NMCP and previous publications on funding, surveillance, and case management to provide a complete overview of the progression of malaria control efforts in Ayeyarwady and how these changes have contributed to the decrease in malaria cases from 2013 to 2017.

## Methods

### Study area

**Figure 1:**
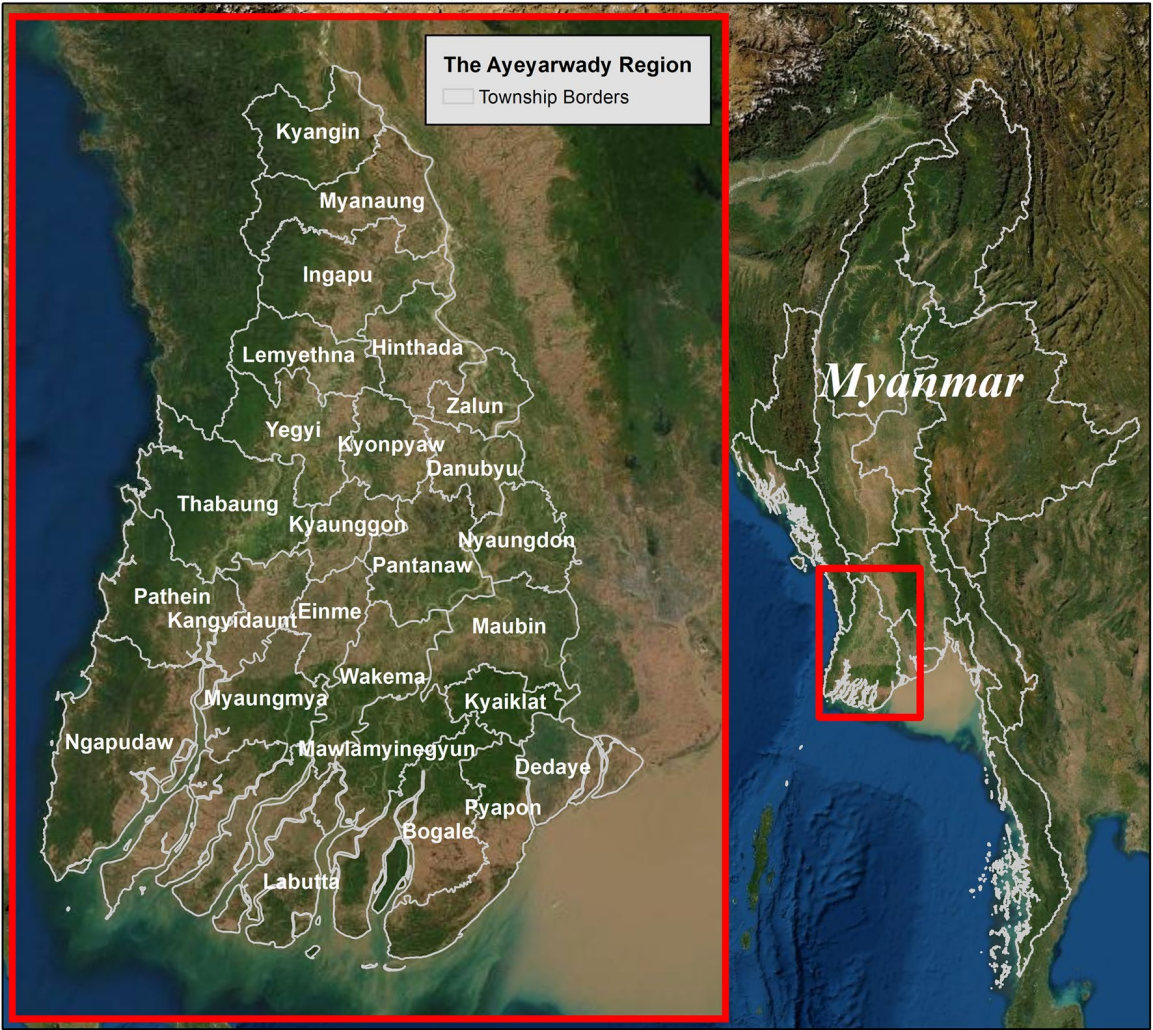
The Ayeyarwady Region of Myanmar. Maps were created using ArcGIS software by Esri. ArcGIS and ArcMap are the intellectual property of Esri and are used herein under license. Copyright © Esri. All rights reserved. For more information about Esri software, please visit www.esri.com. Base map credits: Esri, Digital Globe, GeoEye, i-cubed, Earthstar Geographics, CNES/Airbus DS, USDA FSA, USGS, AEX, AeroGRID, Getmapping, IGN, IGP swisstopo, and the GIS User Community. Software: ArcGIS 10.7.

The Ayeyarwady Region in Myanmar is a delta region bordered to the west by the Bay of Bengal and to the south by the Andaman Sea (Fig. 1). The region is primarily agricultural and the most recent census in 2014 estimated a regional population of 6.2 million that was 86% rural^12, 13^. The settled population is estimated to be relatively stable, though the mobile migrant population varies over time (M. Tun, personal communication, 2018; T. Htay, personal communication, 2018). Ayeyarwady has three seasons: summer (March–April), rainy (May–October), and cold season (November-February). Annual rainfall is approximately 2.5 m and the average temperature is 32 degrees Celsius^14^. Ayeyarwady has six administrative districts, twenty-six townships, and 11,792 villages^15^. The health system hierarchy consists of 1,505 health catchment areas with an average of eight villages associated with a primary health facility. Malaria testing, treatment, case notification, and health education is carried out by BHS and ICMVs (who are trained in the public sector). BHS, alongside other township health staff, are responsible for malaria surveillance activities such as case investigation. Distribution of LLINs occurs continuously at health facilities for high-risk individuals and every three years via mass campaign, prioritizing populations with the highest reported transmission strata defined by the VBDC for intervention targeting (first API > 5, then API 1–5, then API < 1)^16^.

### Data sources and availability

Data from Ayeyarwady on malaria incidence, programmatic financing, surveillance, case management, vector control interventions, climate and ecological factors, and policies and guidelines spanning 2013 to 2017 were compiled retrospectively for this analysis. Data on number of malaria treatments given over the time period were not accessible and therefore not explored in this analysis. Explanatory factors included policy changes, financing, changes in surveillance and case management, major training of new and existing staff, vector control interventions, and changes in climate and ecological variables. These data were collected from publications and NMCP reports, as well as via informational interviews focused on technical information regarding changes in policies, guidelines, and procedures (the interviews were on objective data points, not attitudes related to the changes). These interviews were conducted over the phone and in person between August 2018 and March 2019 with Myanmar malaria program officials, including the Assistant VBDC Director, Malaria Assistants, and BHS. Interviews were mainly used to fill any gaps in the documented history and focused on regional-level funding and changes to guidelines, policies, and processes for case management, surveillance, and vector control. Table 1 provides a summary of the sources and availability of the data included in the analysis. Additional information on data availability and definitions is available in Supplementary Table S1.

**Table 1:** Definition, source, spatial resolution, and years of data included in the assessment.

Data	Definition	Source	Spatial resolution	Years available
Population	Estimated inhabitants	MIMU, PMI^6, 17^	Township	2013–2017
Guidelines, policies, and trainings	Guideline changes, policy changes, and trainings for health care workers or VBDC staff that may have impacted malaria transmission	Annual reports, annual reviews, personal communications^3, 5, 11, 14, 18–20^	Region	2013–2017
**Malaria data**				
Malaria cases	Number of malaria cases confirmed via RDT or microscopy	Malaria Information System^21^	Region, Township, PoC type	2013–2017
Malaria tests	Number of patients tested with RDT or microscopy	Malaria Information System^21^	Township, PoC type	2013–2017
ABER	Total malaria tests divided by total population	Calculated from Malaria Information System and Population	Township	2013–2017
**Ecological**				
Temperature	Average annual land surface temperature (daytime, in degrees Celsius)	MODIS derivative^22^	1 km^2^	2013–2017
Precipitation	Atmospheric precipitation (rainfall in mm)	CHIRPS^23^	1 km^2^	2013–2017
Forest loss	Percentage of township covered by forest each year	Hansen/UMD/Google/USGS/NASA^24^	1 arc-second (approx. 30 m)	2013–2017
	Cumulative percentage of forest cover lost in each township since 2000			
**Financing**				
National funding	Funding in USD for malaria control and elimination	World Malaria Reports^25–30^	Country	2013–2017
Regional funding		Annual reports, annual reviews, personal communications^3, 5, 11, 14, 18–20^	Region	2013–2017
**Surveillance**				
Reporting completeness	Number of health facilities and ICMVs that reported data divided by number expected to report (average monthly percentage)	VBDC^5, 18–20^	Township (BHS/ICMV)	2014–2017
**Case management**				
ICMV placements	Number of ICMVs placed in each township	VBDC^5, 18–20^	Township	2013–2017
**Vector control**				
LLIN targeted population and distribution	Total population targeted and number of LLINs distributed	VBDC^19, 31, 32^	Region	2013–2015
	Total population targeted and number of LLINs distributed	VBDC^5, 20, 31, 32^	Township	2016–2017
LLIN coverage	Population covered (total LLINs*1.8) divided by population targeted	Calculated^19, 20, 33^	Townships receiving LLINs	2016–2017

*ABER* Annual Blood Examination Rate, *CHIRPS* Climate Hazards Group InfraRed Precipitation with Station data, *ICMV* Integrated Community Malaria Volunteer, *MIMU* Myanmar Information Management Unit, *MODIS* Moderate Resolution Imaging Spectroradiometer, *PMI* President’s Malaria Initiative, *PoC* Point of Care, *VBDC* Vector-Borne Disease Control Program.

### Data analysis

This was an exploratory analysis. Throughout this analysis malaria cases were defined as all confirmed cases, regardless of species.

Detection biases in the incidence data were evaluated by describing temporal trends of the total number of tests, mean annual blood examination rate (ABER), and mean reporting rates by health facility and ICMV at the township level along with 95% CI.

Secondly, we described epidemiological trends in incidence and test positivity rate at the regional and township levels during the study period (township level means and ranges) using univariate analysis considering one variable at a time. Because the data were not always available for the same time periods, multivariate analysis was not possible. The longitudinal change in annual township-level incidence with and without adjusting for reporting completeness and the longitudinal change in annual test positivity rate was statistically tested using a repeated measure Poisson regression model for a count outcome (cases) with an offset term of log population and an adjustment for repeated measures within townships to account for correlation within the townships, implemented using a General Estimating Equation (GEE) procedure:

1 $$log\left({cases} \right)\; \sim \;{\text{log}}\left({{\text{population}}} \right) + \beta_{0} + \beta_{{1}}\, *\,{\text{year }} + {\text{cluster}}\;\left( {{\text{township}}} \right)$$

2 $$log\left({cases} \right)\; \sim {\text{log}}\left({{\text{population}}} \right) + \beta_{0} + \beta_{{1}}\, *\,{\text{year}} + \beta_{{2}} \,*\,{\text{reporting }} + {\text{cluster}}\;\left( {{\text{township}}} \right)$$

3 $$log\left({cases} \right)\; \sim {\text{log}}\left({{\text{tests}}} \right) + \beta_{0} + \beta_{{1}} \,*\,{\text{year}} + {\text{cluster}}\;\left( {{\text{township}}} \right)$$

A spatial correlation was not incorporated in the models because there was no expected violation of independence between townships for the covariates included in the model.

To assess the association between the environmental variables and the epidemiological trends, annual township level means and ranges were calculated for temperature, precipitation, and percentage of total area forested for 2013 through 2017.

We explored the relationship between incidence and cumulative percentage of forest cover lost since 2000 using a GEE model with a Poisson distribution for a count outcome (cases), population exposure, and an adjustment for repeated measures within townships. The model was limited to Ngapudaw, Pathein, and Thabaung, which are located in the forest fringe and had the highest number of malaria cases in Ayeyarwady during the study period.

4 $$log\left( {cases} \right)\; \sim {\text{log}}\left( {{\text{population}}} \right) + \beta_{0} + \beta_{{1}} *{\text{year}} + \beta_{{2}} *{\text{forest}} + {\text{cluster}}\;\left( {{\text{township}}} \right)$$

Funding and case management were descriptively assessed at the regional level. Fiscal data on the details of malaria funding in Ayeyarwady were not available, so national level data were explored. Testing and number of ICMVs placed were used to describe case management system change. To probe into ICMV performance, mean township-level ABER in townships with and without ICMVs was compared descriptively and was statistically tested using a Poisson model (ICMV = 1 for townships with ICMVs and 0 otherwise).

5 $$log\left( {tests} \right)\; \sim {\text{log}}\left( {{\text{population}}} \right) + \beta_{0} + \beta_{{1}} *{\text{year}} + \beta_{{2}} *{\text{ICMV}} + \beta {3}*{\text{year}}*{\text{ICMV}} + {\text{cluster}}\;\left( {{\text{township}}} \right)$$

Finally, we described the regional level distribution and coverage of LLINs and explored the trend in incidence in townships that received LLINs and those that did not. The change in incidence over time was compared between townships that received LLINs in 2016 or 2017 and townships that did not.

Statistical analysis was performed using Stata S/E 14. Maps were created using ArcGIS software by Esri. ArcGIS and ArcMap are the intellectual property of Esri and are used herein under license. Copyright © Esri. All rights reserved. For more information about Esri software, please visit www.esri.com. Base map credits: Esri, Digital Globe, GeoEye, i-cubed, Earthstar Geographics, CNES/Airbus DS, USDA FSA, USGS, AEX, AeroGRID, Getmapping, IGN, IGP swisstopo, and the GIS User Community. Software: ArcGIS 10.7.

## Results

### Surveillance and data reliability

Testing rates and reporting completeness were quantitatively assessed in order to establish the validity of the available case data used in all following analyses and the validity of any determined patterns of case burdens over time. Testing and case counts were positively correlated (R = 0.64). The total number of tests conducted in Ayeyarwady increased three-fold, from 36,383 in 2013 (n = 20 townships) to 111,655 in 2017 (n = 26 townships); this drove an increase in mean township-level ABER from 0.81% (Standard Deviation [SD] 0.91) to 1.91% (SD 1.25). On average, testing increased by 29% each year (incidence rate ratio [IRR] = 1.29, 95% CI 1.19–1.41, p < 0.01) (Table 2).

**Table 2:** Summary of surveillance and data reliability changes between 2013 and 2017.

Outcome Variable	IRR	Confidence intervals	P Value
Annual change in malaria testing	1.29	1.19–1.41	< 0.01
Annual change in incidence of malaria per 1,000	0.69	0.64–0.75	< 0.01
Annual change in test positivity rate	0.53	0.44–0.64	< 0.01

**Figure 2:**
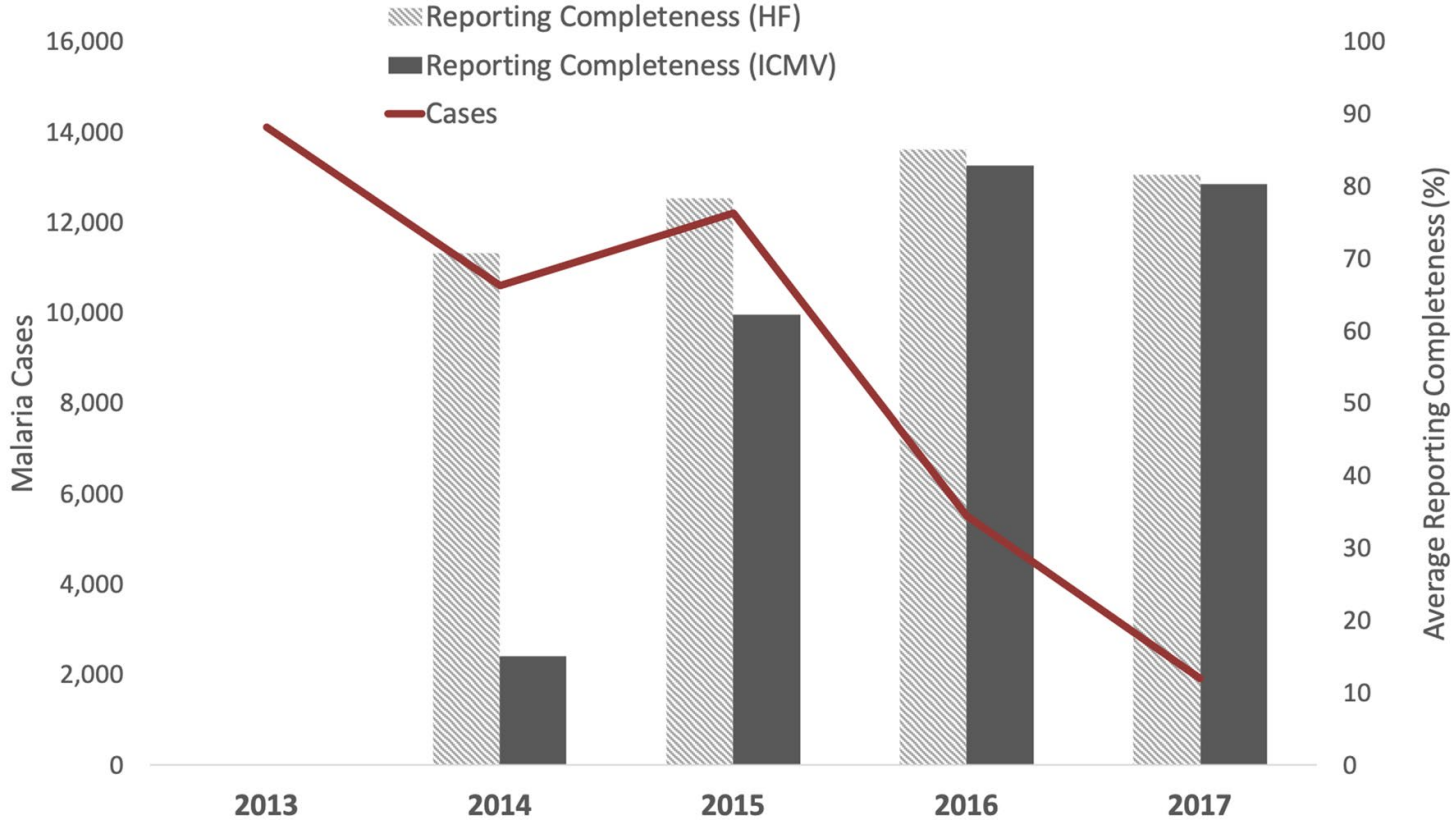
Number of malaria cases and reporting completeness in Ayeyarwady, Myanmar, 2013 to 2017 (reporting completeness data were not available for 2013). Acronyms: Health Facility (HF), Integrated Community Malaria Volunteer (ICMV).

Reporting completeness was also reviewed in order to determine whether any patterns in case burdens were due to actual transmission patterns or artifacts of reporting. Mean township-level health facility reporting improved from 70.77% in 2014 (SD 21.04) to 81.59% in 2017 (SD 12.6) (2013 data were not available) (Fig. 2). Reporting among ICMVs improved from 15.04% in 2014 (SD 17.64) to 80.32% (SD 21.41) in 2017. The increase in reporting completeness was achieved by conducting trainings for over 4,000 BHS across Ayeyarwady to transition from aggregated health facility reports to individual case-based reporting. Additionally, in January 2017, township-level staff were trained to enter case data electronically, enabling rapid data entry and providing access to summarized case and testing data for township-level staff. These findings indicate that there was a high (and increasing) rate of testing and reporting completeness in Ayeyarwady over the period included in this study.

**Figure 3:**
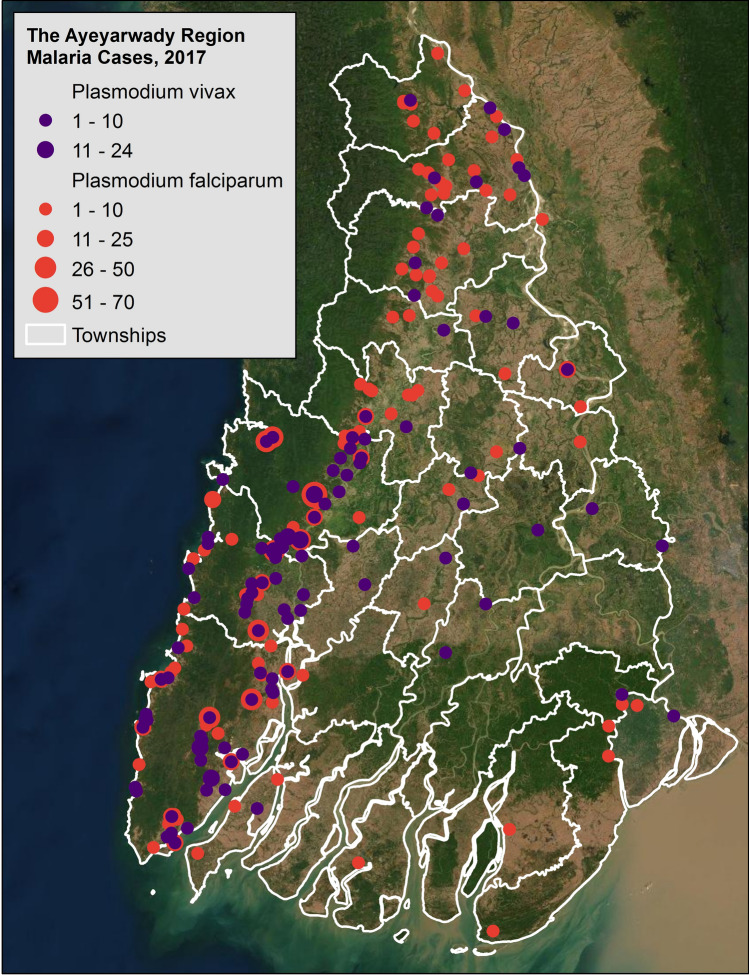
Malaria cases in Ayeyarwady at the health facility level, 2017. Maps were created using ArcGIS software by Esri. ArcGIS and ArcMap are the intellectual property of Esri and are used herein under license. Copyright © Esri. All rights reserved. For more information about Esri software, please visit www.esri.com. Base map credits: Esri, Digital Globe, GeoEye, i-cubed, Earthstar Geographics, CNES/Airbus DS, USDA FSA, USGS, AEX, AeroGRID, Getmapping, IGN, IGP swisstopo, and the GIS User Community. Software: ArcGIS 10.7.

In 2017, the latest year of the study period, there were 1,919 cases of malaria (reported through the public sector) and one death due to malaria in Ayeyarwady. Of reported cases, 73% were *P. falciparum* and 27% were *P. vivax* (Fig. 3), 74% of cases were male, and 87% of cases were 15 years or older. Malaria cases peaked in June. As is historically typical, malaria burden was highest in the forested range on the western side of Ayeyarwady.

**Figure 4:**
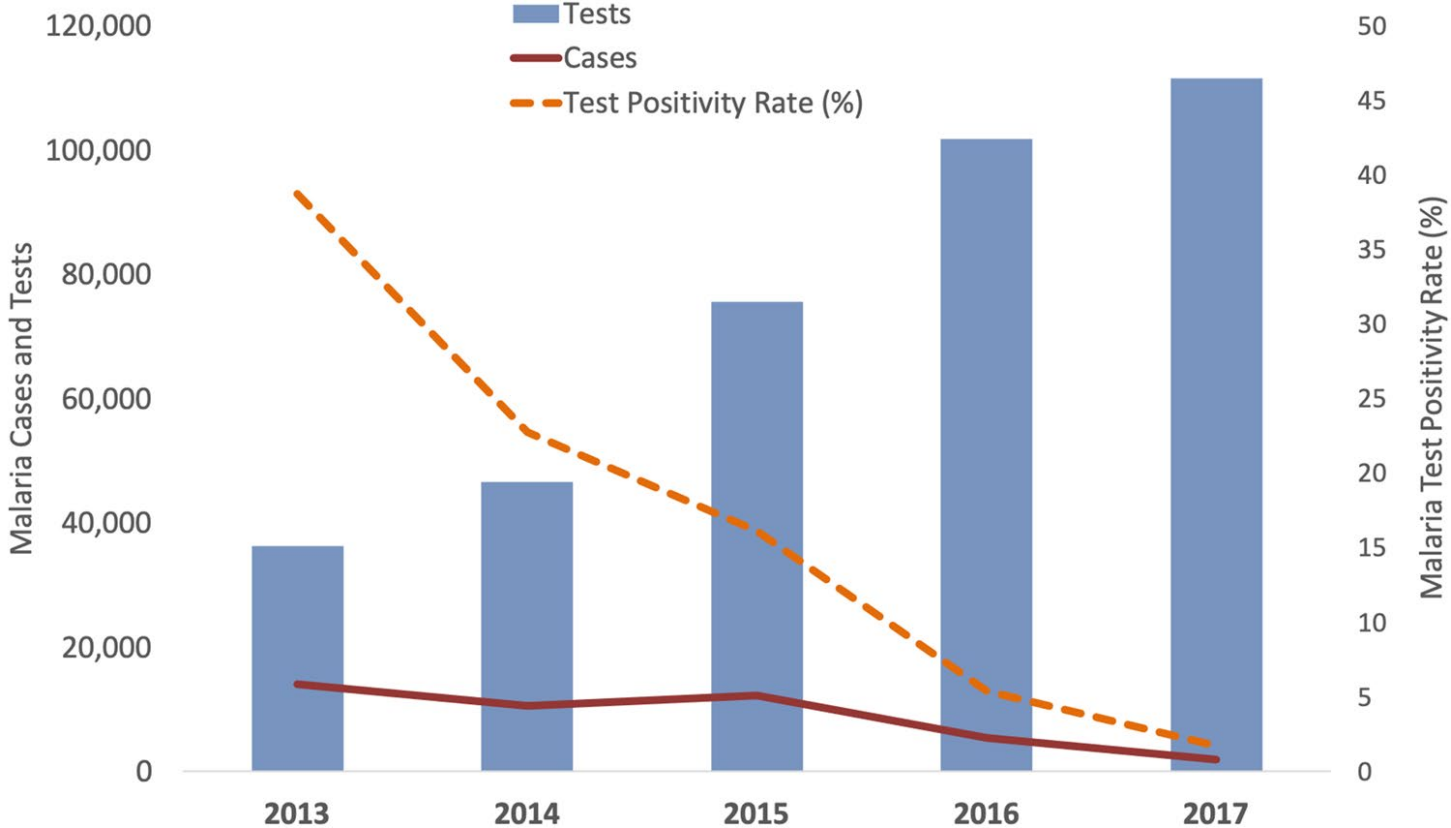
Number of malaria tests, number of malaria cases, and malaria test positivity rate in Ayeyarwady, Myanmar, 2013 to 2017.

Between 2013 and 2017, confirmed malaria cases in Ayeyarwady dropped 86% from 14,099 (n = 20 townships) to 1,919 (n = 26 townships). Mean township-level incidence per 1000 population (for 20/26 townships with non-missing case values in 2013) declined from 3.03 (SD 4.59) in 2013 to 0.34 (SD 0.79) in 2017, decreasing 31% per year on average (IRR = 0.69, [95% CI 0.64–0.75], p < 0.01). In Ayeyarwady, 39% of tests were positive in 2013, compared with 1.7% of tests in 2017 (Fig. 4). Mean township-level test positivity rate (TPR) declined from 29.63% in 2013 (SD 0.23) to 1.05% in 2017 (SD 0.018), a statistically significant average annual decline of 47% each year (IRR = 0.53, [95% CI 0.44–0.64], p < 0.01). After adjusting for reporting completeness among health facilities, confirmed cases still decreased by 36% each year (IRR = 0.64, [95% CI 0.57–0.72], p < 0.01).

### Environmental factors

**Figure 5:**
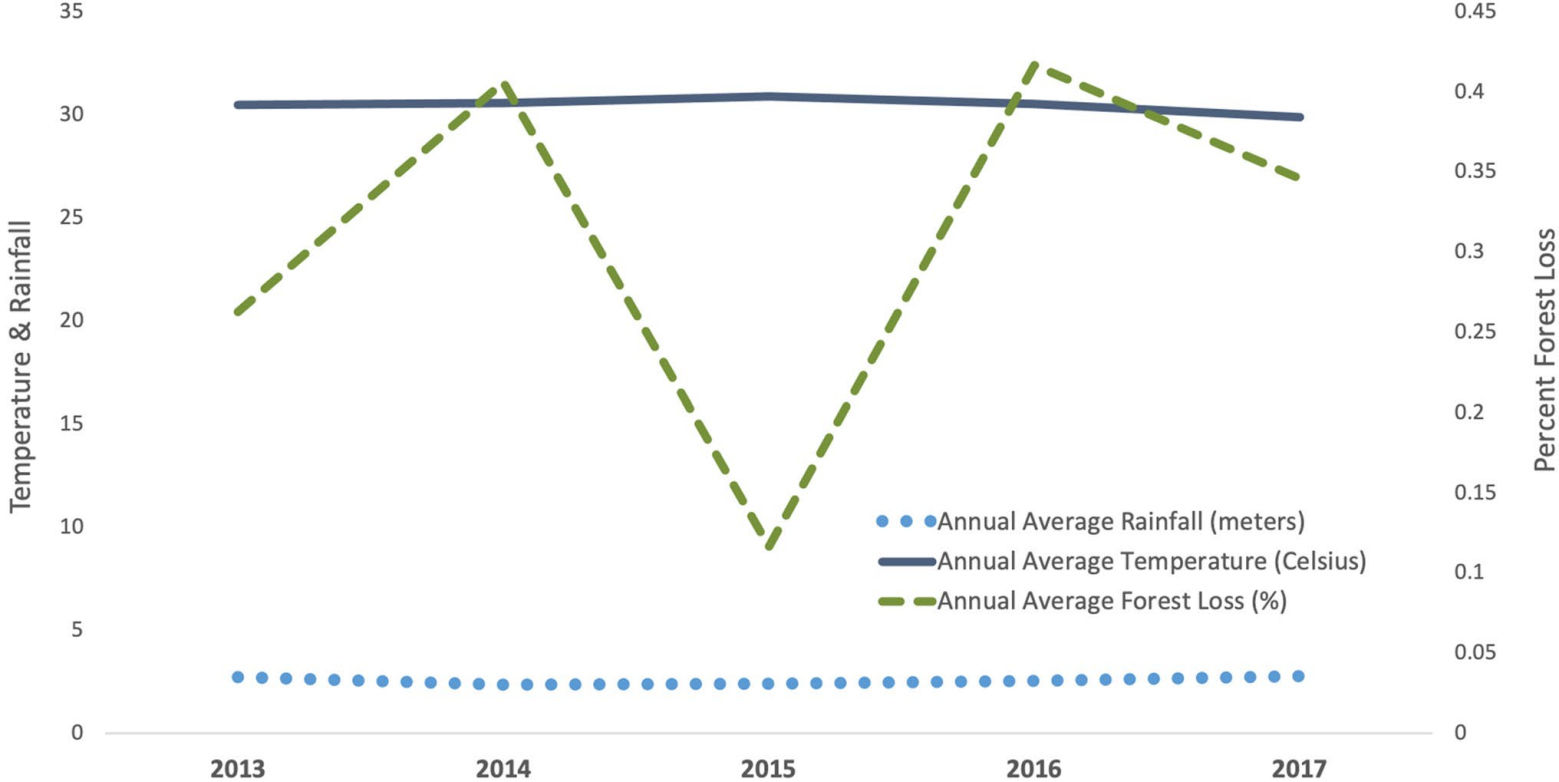
Annual average forest loss (%), annual average temperature (Celsius), and annual average rainfall (meters) in Ayeyarwady between 2013–2017.

Annual mean township-level temperatures ranged between 28.49 degrees Celsius and 32.68 degrees Celsius during the study years with no observed outliers when reviewed descriptively. Within townships, mean annual temperature deviated the least in the eastern township of Kyaiklat (0.7 degrees Celsius, range 28.89- 29.59 degrees Celsius) and the most in the northern township of Ingapu (1.33 degrees Celsius, range 30.64–31.97 degrees Celsius). Mean temperature across townships was highest in 2015 (30.87 degrees Celsius, SD 1.04) and lowest in 2017 (29.87 degrees Celsius, SD 0.92) (Fig. 5).

As with temperature, no distinct outliers were visible in the annual mean township-level rainfall data, which ranged from 1270 to 4221 mm. Nyaungdon, on the region’s eastern border, varied the least (1914–2233 mm) while Ngaputaw, in the western forest fringe, varied the most (3511–4221 mm). Across townships, mean rainfall was lowest in 2014 (2359 mm, SD 566) and highest in 2017 (2749 mm, SD 567).

**Figure 6:**
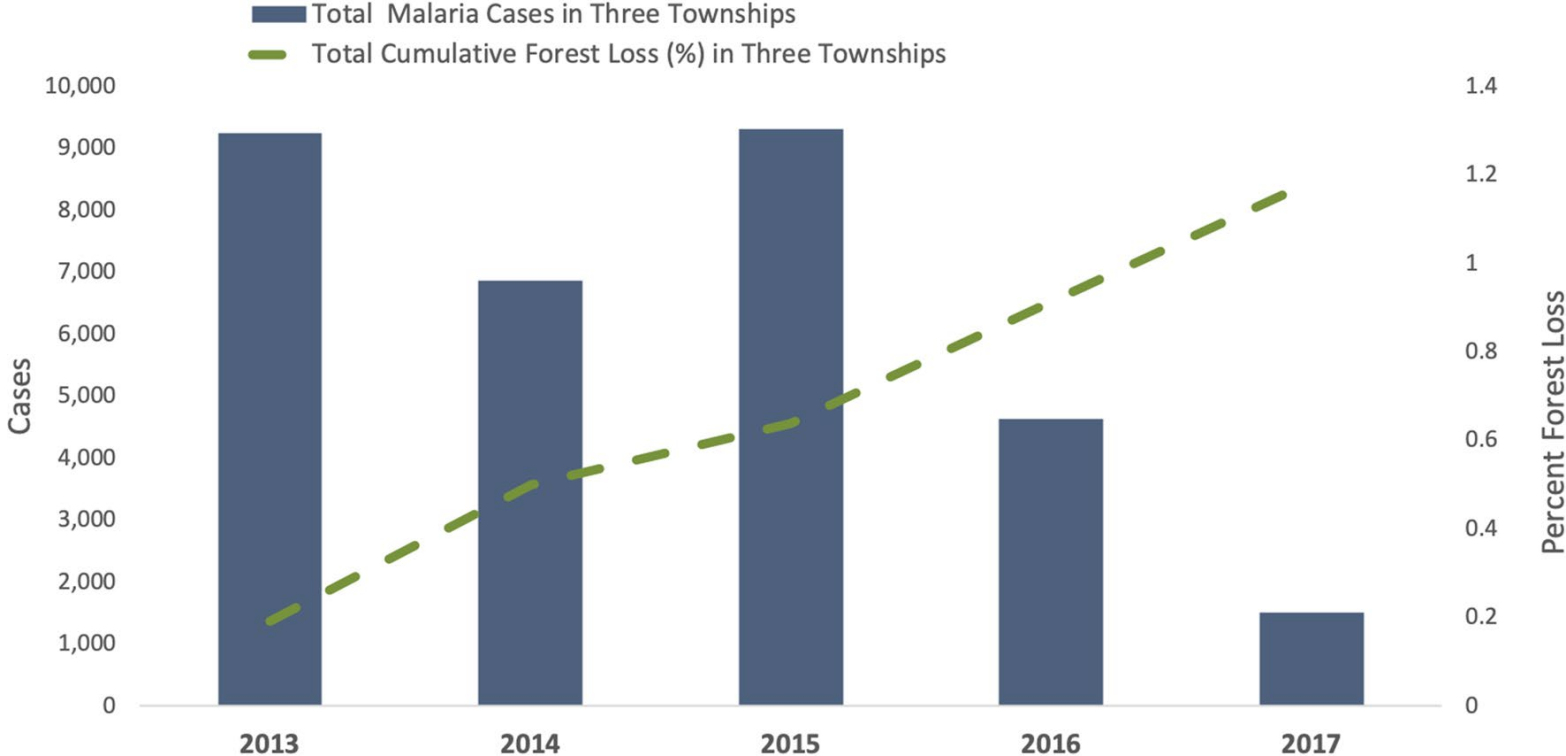
Sum of the annual number of malaria cases and sum of the annual cumulative percentage of forest loss in the three highest malaria burden townships in Ayeyarwady between 2013 and 2017. The three townships are Ngapudaw, Pathein, and Thabaung.

Ayeyarwady has experienced substantial annual declines in the township-level percentage of land covered by forest from 15.23% (SD 18.22) in 2013 to 13.95% (SD 17.71) in 2017. Three townships along the southern forest fringe of western Ayeyarwady (Ngapudaw, Pathein, and Thabaung) accounted for 73% of cumulative forest loss in the region from 2013 to 2017 (almost 120 million square meters). Cases in Ayeyarwady have become increasingly more focalized to the same three townships, accounting for 66% of the region’s cases in 2013 and 79% by 2017. In these three townships, malaria cases per year were inversely related to cumulative percentage forest loss since the base year of 2001. Each percentage point increase in cumulative forest area lost was associated with a decrease in the rate ratio of cases by a factor of 0.92 after adjusting for year (adj IRR = 0.92, 95% CI 0.90–0.93, p < 0.01) (Fig. 6). The relationship between malaria cases and forest loss in the remaining townships was not significant.

### Malaria interventions

**Figure 7:**
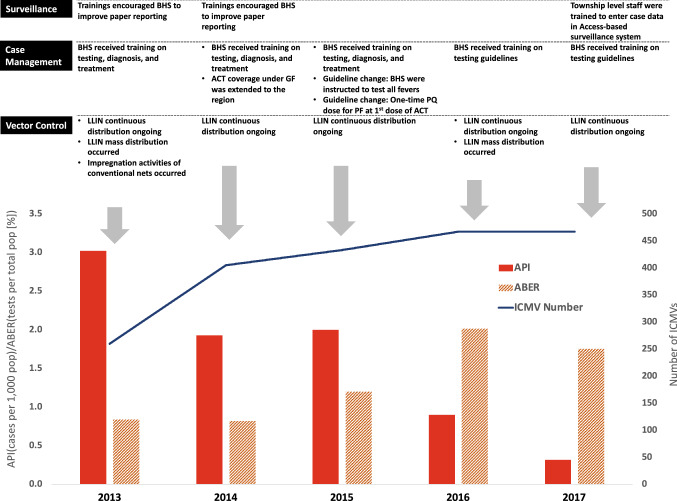
Timeline of significant events in malaria surveillance, case management, and vector control compared to changes in annual parasite incidence (API), annual blood examination rate (ABER), and number of Integrated Community Health Volunteers (ICMVs). Additional acronyms: Basic Health Staff (BHS), Long-lasting insecticide treated nets (LLINs), Global Fund to Fight AIDS, TB, and Malaria (GF), artemisinin-based combination therapy (ACT), primaquine (PQ), integrated community health volunteer (ICMV).

Figure 7 shows the timeline of major events between 2013 and 2017 in the categories of malaria surveillance, case management, and vector control.

#### Funding

Total funding for malaria in Myanmar was over United States Dollars (USD) 22.4 million in 2013, following a rapid increase beginning in 2011^25–30^. National financing for malaria reached USD 79.7 million in 2017 (67% from The GF)^30^. Reported national domestic financing for malaria programs has also increased, from less than USD 1 million in 2013 (4% of total funds) to USD 6.6 million (8% of total funds) in 2017^25, 30^. Ayeyarwady has received GF funding support from since 2011 for activities such as training BHS and malaria volunteers, for ICMV payments, and commodities such as RDTs, laboratory materials, anti-malarial drugs, LLINs, and insecticide tablets for treating conventional nets. Between 2015 and 2017, investments were made by Population Services International (PSI) and Myanmar Medical Association (MMA) for multiple private sector activities^3, 5, 11, 14, 18–20^. The first consisted of investing in general practitioners and the second on mobile clinic activities.

#### Community case management

From 2013 to 2017, considerable investments were made to improve case management in Ayeyarwady and expand access to diagnosis and treatment at the community level. ICMVs were placed in the villages with at least 150 people and in the highest annually defined risk strata based on API distribution and vulnerability [M. Tun, personal communication, 2018]. Beginning in 2015, travel allowance was provided to support the ICMVs. Between 2013 and 2017, the ICMV program expanded from 260 to 467 ICMVs, and volume of tests performed by ICMVs increased from 3,231 in 2013 to 18,923 in 2017. In townships with ICMVs in 2017, the ABER increased from 1.38% in 2013 (SD 0.96) to 2.90 in 2017 (SD 1.27). Townships without ICMVs in 2017 also increased ABER from 0.24% in 2013 (SD 0.32) to 1.18% in 2017 (SD 0.53). Townships with ICMVs had 26% higher increases in testing than those without (IRR = 1.26 95% CI 1.12–1.41, p < 0.01). As the number of ICMVs in the region increased, the percentage of cases captured by ICMVs increased from 7.5% of cases in 2013 to 38.6% of cases in 2017.

Policy changes in Ayeyarwady between 2013 and 2017 improved malaria care; in 2015 BHS and ICMVs were instructed to test all fever cases for malaria, were trained in the proper application of ACT treatment, and started administering primaquine doses at the beginning of the ACT course (instead of at the end of the treatment course) to ensure compliance to treatment^34^. In addition, an electronic logistics supply management system, mSupply, was initiated in Ayeyarwady in 2016 to assist with stock management and reduce stock-outs of diagnostic and treatment commodities at the regional level (it does not assist with stock management and stock-outs at the township or health facility level).

#### Vector control

Mass distribution of LLINs occurred in 2013 and 2016–2017, in addition to continuous distribution at public health facilities to patients at risk, including those who work in the forest, are pregnant, or have tested positive for malaria. Township-level resolution on LLIN targeting and delivery across the region was available for 2016 and 2017. Eight townships received LLINs in 2016 or 2017 (Ingapu, Kyangin, Lemyethna, Myanaung, Ngaputaw, Pathein, Thabaung, and Yegyi), in alignment with 2016–2020 National Strategic Plan targeting to townships with API greater than 1 in 2015. In 2016, 204,439 LLINs were distributed to a target population of 431,016 (87.39% coverage); in 2017, 183,460 more LLINs were delivered to a target population of 371,486 (88.89% coverage).

In these eight townships, mean township-level incidence (per 1,000) declined from 6.87 (SD 5.32) in 2013 to 1.03 (SD 1.21) in 2017 (a decrease of 85%). Malaria incidence also declined in the remaining 18 townships that did not receive LLINs, from 0.47 (SD 0.71) in 2013 to 0.03 (SD 0.03) in 2017 (a decrease of 94%).

## Discussion

Reported cases of malaria decreased 86% between 2013 and 2017 in Ayeyarwady, Myanmar, a drop that suggests a real decline in transmission as it was not explained by detection bias (low reporting or testing). In this exploratory analysis, we found that the reasons behind this drastic decline were likely multifactorial and occurred in tandem. While it is difficult to quantify the impact of overlapping interventions, it is important to explore the disease control environment within which this impressive decline in malaria has occurred. In this analysis, we gathered quantitative and qualitative information on environmental factors and malaria control interventions and assessed these factors in relation to the changing malaria epidemiology in Ayeyarwady. Table 3 summarizes the key findings of this analysis.

**Table 3:** Summary of possible explanatory categories for the decline of malaria in Ayeyarwady.

	Summarized main findings by category	Potential implications for the decline of malaria
Ecological factors	- In the three townships with the largest malaria caseloads, greater forest loss was associated with greater decline in cases (adj IRR = 0.92, 95% CI 0.90–0.93, p < 0.01) - No major outliers were observed in mean annual township-level daytime temperature and precipitation between 2013 and 2017	Forest loss could have directly or indirectly contributed to declines in malaria in the three highest-burden townships; however, it is unlikely that malaria transmission trends are explained by climate during this period.
Funding	- National level funding for malaria increased during 2013—2017	Additional funds were available for anti-malaria activities and commodities.
Surveillance	- A shift from aggregate to case-based reporting of cases occurred - Trainings on reporting were held for BHS and ICMVs - Completeness of township-level health facility and ICMV reporting improved	The decrease in malaria cannot be explained by reporting bias, as reporting completeness improved during the study period. During this period, the program began to have a more granular understanding of the malaria burden in the region. The trainings likely contributed to the improved reporting completeness rates and thus higher-quality data. Accurate data can lead to improved understanding of malaria transmission in the region and improved targeting of resources.
Case Management	- Increased testing (IRR = 1.29, 95% CI 1.19–1.41, p < 0.01) - Increases in numbers of ICMVs placed in villages - Increases in the number of tests conducted by ICMVs and townships with ICMVs had 26% higher increases in testing than those without (IRR = 1.26 95% CI 1.12–1.41, p < 0.01) - Guidelines changed to encourage the testing of all fevers - Trainings on treatment guidelines were held - ABER increased	The decrease in malaria cannot be explained by detection bias because ABER improved during the study period. Improved access to testing and diagnosis may have led to increased detection of malaria cases and, in conjunction with improved treatment for malaria, may have decreased onward transmission and reduced incidence.
Vector Control	- Mean township-level incidence declined in townships that received LLINs and in townships that did not receive LLINs	LLIN distribution may have contributed to the reduction in malaria transmission in higher transmission areas but cannot explain declines in malaria across Ayeyarwady.

Three ecological variables (temperature, rainfall, forest cover) were assessed for any obvious anomalous events that could affect transmission dynamics and explain the decline in cases. While no major deviations in temperature or rainfall were observed, there was a noticeable inverse relationship between cumulative annual forest loss and malaria burden when analysis was restricted to the three highest burden townships, which lie along the western forest fringe area of Ayeyarwady. This result introduces the possibility that environmental change through deforestation in Ayeyarwady may play a role in the decline of malaria, although possibly only when malaria burden is high or when there is a large change in forest cover (these three townships represented 73% of the forest loss in Ayeyarwady). The result at township level may also be incidental because malaria declined in townships that did not have significant forest loss, and a burden reduction assessment conducted in other regions of Myanmar in 2019 did not find an association between deforestation and malaria caseload^6^. However, in Ayeyarwady, forest cover has declined by approximately 2% per year, twice the national average, due to logging, mining, conversion to plantations, and other agricultural activities^35, 36^. Additional studies are required to elucidate whether there is a relationship between deforestation and malaria incidence in Myanmar and in the GMS to better understand the mechanisms driving any correlation^37, 38^. Our findings indicate forest loss is likely not the only relevant factor related to the decline of malaria in Ayeyarwady.

During the study period, changes in malaria control occurred in all sectors we examined: funding, surveillance, case management, and vector control. The central and local governments in Myanmar, in collaboration with partners, have implemented several interventions with the goal of halting malaria transmission in the region; the evidence collected here suggests that these efforts have been successful. During the study period, increased funding was available for interventions and commodities that have been shown to reduce malaria in other contexts. Previous studies on malaria in Myanmar and elsewhere have noted that increased funding for the health sector and for malaria activities coincides with declines in malaria burden^7, 39^.

Malaria surveillance improved during the study period through trainings conducted to increase reporting and a shift from an aggregate to a case-based reporting system. The finer detail in the epidemiology and location of malaria cases in Ayeyarwady allowed for more data-driven and targeted interventions (for example, the placement of ICMVs in villages with high risk). Improving surveillance is defined by the WHO as a key malaria intervention^40^. Although there is limited literature on the direct effects of improved malaria surveillance on declining malaria, countries that have achieved malaria elimination have relied on robust surveillance systems^41, 42^.

Between 2013 and 2017, the Ayeyarwady VBDC also focused on improving access to quality malaria case management. The program invested in updated guidelines and a well-trained healthcare workforce and a 2019 assessment conducted by USAID found that person-days of malaria training provided were associated with decline in malaria cases^6^. Community care in highest-risk areas was made more available by targeted deployment of new ICMVs in these areas; a study comparing ICMVs to BHS in Myanmar found that ICMVs were more accessible to children and women for malaria screening and treatment^43^. In fact, integrated community malaria volunteers reported 38.6% of all cases in Ayeyarwady in 2017, underscoring the utilization of community case management services. Improvements to detection and treatment are likely involved with reducing malaria transmission as fewer parasites are in circulation.

Finally, control of mosquito vectors through the distribution of LLINs continues to be a main component of the Myanmar VBDC’s malaria elimination efforts. During the 2016–2017 mass campaign, coverage was programmatically successful at nearly 90%; actual coverage may be even higher due to rolling distribution. Both townships without LLINs and those that received LLINs in 2016 and 2017 experienced a decline in malaria incidence during the study period; those without LLINs experienced a greater proportional decline in incidence (94% versus 85% decline). LLINs may have averted many cases during the study period; in 2017, townships with LLINs reported over 10,600 fewer cases than in 2013, while in the eighteen townships with no LLINs, the drop was approximately 1,500 cases. The comparison between these groups is not truly interpretable given the heterogeneity in transmission at the beginning of the study period (townships that received LLINs had a much higher incidence in 2013 [6.9/1000 compared with 0.3/1000]) but the declines in townships with LLINs suggest a potential protective benefit of the intervention. The effectiveness of LLINs on malaria transmission is well established in general^44^; however, research into malaria risk factors in the GMS region^45, 46^ and Ayeyarwady specifically [Dunning et al*.*, *manuscript in preparation*] has determined that the population at risk is more likely to be forest-sleeping individuals who may not be protected by bed nets. Interventions are being deployed to reach these higher-risk “forest-goers” both in Myanmar and the wider GMS, such as supplying people who frequent the forest with hammock LLINs that they can sleep under when spending the night in the forest^47–49^. Further research is required to determine whether hammock LLINs would be acceptable and effectively used in the Myanmar context.

This study had several limitations. First, data completeness was limited by availability. Private sector data on cases and testing were not available; a report from 2017 estimated that approximately 5% of malaria cases in Ayeyarwady were captured by the private sector that year^50^. Township-level LLIN data were available for 2016 and 2017, but were only available at the regional level for prior years. Monthly epidemiological data would improve the study by allowing for more robust exploration of fluctuations in climate variables; the relationships between climatic factors and malaria have been explored in numerous contexts and will become increasingly important to understand as climates change^51–53^. Additionally, limited data were available on rates of treatment completion and health seeking behavior in Ayeyarwady, which prevented any analysis of these factors. A second limitation was the inability to infer causality based on the observed associations between the different factors and malaria because this was not a randomized control trial and because the individual analyses did not account for other factors. This challenge is further highlighted by the heterogeneity in geographic and temporal scale of the available retrospective data, which necessitated a variety of outputs rather than a single exploratory model. Going forward it may be possible to gain insight into several of these indicators that are now being captured at a more granular level in Ayeyarwady. This may enable multivariable modeling with larger timespans and numbers of outcome data points, controlling for confounding factors, and disentangling the relative impact of each intervention. Qualitative information collected on guidelines and changes in malaria procedures provided a more nuanced description and understanding of malaria elimination efforts in Ayeyarwady as there are numerous potential factors related to the decline and the interventions are often-changing. However, qualitative information collected via interviews may be subject to recall bias. Finally, there were potentially confounding factors that were not assessed in this study including socio-economic status, vulnerability of the populations (e.g., in relation to demographics, occupational hazards, and conflicts), and mobility of the at-risk population.

## Conclusion

The quantitative and qualitative findings in this study suggest there has been a decline in malaria transmission in Myanmar’s Ayeyarwady region that coincides with implementation of the government’s multi-pronged malaria elimination strategy and with a period of deforestation. Although it is difficult to measure the impact of changes in malaria control guidelines and interventions, a large increase in malaria control funding has led to increased investment in training, case management practices and commodities such as LLINs and RDTs. This study suggests that the improvements in funding, surveillance and case management and the deployment of interventions for malaria elimination have played valuable roles in decreasing the malaria burden in Ayeyarwady.

## Supplementary Information

Supplementary Information.

## Data Availability

The datasets generated and/or analyzed during the current study are not publicly available due to data protection from the Vector Borne Disease Control Program in Myanmar but can be available from the corresponding author with country approval.

## Abbreviations

ABER: Annual Blood Examination Rate
ACTs: Artemisinin-based combination therapies
API: Annual Parasite Index
BHS: Basic Health Staff
CHAI: Clinton Health Access Initiative
CHIRPS: Climate Hazards Group InfraRed Precipitation with Station data
CI: Confidence interval
GEE: Generalized estimating equation
GF: The Global Fund to Fight AIDS, Tuberculosis and Malaria
GMS: Greater Mekong Subregion
ICMV: Integrated Community Malaria Volunteer
IEC: Information, Education and Communication
IRR: Incidence rate ratio
ITN: Insecticide treated net
LLIN: Long-lasting insecticide treated net
MIMU: Myanmar Information Management Unit
MIS: Malaria Information System
MMA: Myanmar Medical Association
MODIS: Moderate Resolution Imaging Spectroradiometer
NMCP: National Malaria Control Program
PMI: President’s Malaria Initiative
PoC: Point of Care
PSI: Population Services International
RDT: Rapid diagnostic test
TPR: Test positivity rate
USAID: United States Agency for International Development
USD: United States Dollar
VBDC: Vector Borne Disease Control Program
WHO: World Health Organization
